# Management of Cosmetic Embarrassment Caused by *Malassezia* spp. with Fruticose Lichen *Cladia* Using Phylogenetic Approach

**DOI:** 10.1155/2013/169794

**Published:** 2013-08-29

**Authors:** Anand Pandey, Rohit K. Mishra, Amit K. Tiwari, Awadhesh Kumar, A. K. Bajaj, Anupam Dikshit

**Affiliations:** ^1^Biological Product Laboratory, Department of Botany, University of Allahabad, Allahabad 211002, India; ^2^Department of Horticulture and Medicinal Plants, Mizoram University, Aizawl 796004, India; ^3^Department of Dermatology, Moti Lal Nehru Medical College, Allahabad 211002, India

## Abstract

During anti-*Malassezia* screening of plants by CLSI broth microdilution method, *Cladia aggregata* (Swartz) Nyl. (family Cladoniaceae), a fruticose lichen from Sikkim (northeast Himalayan region), has been found effective at minimum inhibitory concentrations (mg/mL) of 2.72, 0.63, and 1.28 against yeast-like fungi namely, *M. furfur, M. globosa* and *M. sympodialis,* respectively. These test pathogens are responsible for pityriasis versicolor (PV) and seborrheic dermatitis (SD) in humans. We tried to establish the reason for variable MICs against various *Malassezia* spp. using bioinformatical tools, thereby reducing the cost of the experimentation. This is the first report on anti-*Malassezia* activity of *C. aggregata* and thus can serve as a potential source for the development of cosmaceuticals.

## 1. Introduction

Unicellular yeast like fungus *Malassezia* is responsible for causing pityriasis versicolor (PV) and dandruff, which manifests as seborrheic dermatitis (SD) in its severe form in humans (as well as animals) causing physical discomfort and cosmetic embarrassment globally. Hypo- or hyper-pigmented skin on the seborrheic areas of the body characterizes the onset of PV. The symptoms of dandruff can range from mild scaling to fine patchy scales attributed to hyperproliferation of the scalp epidermis, as judged by cell turnover studies and the presence of parakeratotic nuclei present in the shed flakes and the stratum corneum [1, 2]. The widespread occurrence of dandruff can be considered physiological because of the critical maturation processes owing to desquamation of the skin surface arising from the continuous separation of scaly layers of the stratum corneum [3, 4].

French scientist Malassez [5] originally identified *Malassezia*. Later on, Raymond Sabouraud [6] identified a dandruff causing organism in 1904 and named the fungus as “Pityrosporum malassez” in honour of the pioneering work of the French scientist. Further research revealed the strains to be the same at species level and name *Malassezia* was given to the fungus and classified the taxa. Lipophilic *Malassezia *is a common mycoflora of human skin, especially the upper sebaceous parts such as hair which has high sebum excretion [7, 8]. Dandruff is a very common problem worldwide, but in temperate and tropical countries, temperatures are high and people sweat a lot in the summer, providing favourable conditions to the pathogen. As teenagers generally perspire more in comparison to older persons, there is a high chance of proliferation of *Malassezia* in teenagers during summer [9]. Presently, about 14 spp. of *Malassezia* are known [10]. Classical *Malassezia furfur *in scales from the disease PV consists of spherical yeasts, 2.5–8 *μ*m in diameter, producing buds from a narrow base, associated with short filaments which are often distorted and angular [11]. Biochemical investigations showed that azelaic acid produced by *Malassezia* spp. is repressive to neutrophils [12] and is a competitive inhibitor of tyrosinase, a key enzyme in melanogenesis [13], suggesting that azelaic acid may play an important role in abnormal skin pigmentation associated with PV.

All these findings have opened the pathway for understanding disease development in human beings with baseline information about *Malassezia* spp. The use of traditional medicines for curing skin ailments in the world of dermatology has a long historical backdrop. Recent researches have revealed that herbal products have better antifungal efficacy and less or negligible undesirable effects on human beings as compared to chemotherapeutic agents [14]. The changing environmental setting and its prejudicial impact on human health have also stirred scientists to search for nonconventional methods of treatment of various maladies.

Lichens are composite organism consisting of two distinct and dissimilar components: the photobiont and the mycobiont. Later one, being the dominant partner, lichens are taxonomically treated as a class of fungi. The diversity of lichens is maintained by approximately 18,800 recognized species [15]. In comparison with the higher taxa of medicinal and aromatic plants (mainly angiosperms), they have been less explored in the medicinal world, and this portends a very wide scope for a novel search among them for potential agents against *Malassezia*.

*Cladia aggregata* (Swartz) Nyl., a fruticose lichen spp. belonging to the family Cladoniaceae, exhibits good growth in mesic habitats of temperate Himalayas with their following taxonomic characteristics. The primary squamulose thallus bears podetia with elliptical perforations in cortex, 3–7 mm long up to 2 mm in diameter. The podetia have yellow to pale brownish younger parts and dichotomous or sympodial branching in older parts with shiny brown texture. Barbatic acid is the main active compound in addition to other substances such as the stictic, norstictic, and fumarprotocetraric acids, whose occurrence and percentage depend on the area where the lichen is found. Barbatic acid and usnic acid are reported as efficient against microorganisms, cancer cells, and tumors [16, 17].

## 2. Materials and Methods

### 2.1. Test Pathogens

**Figure 1:**
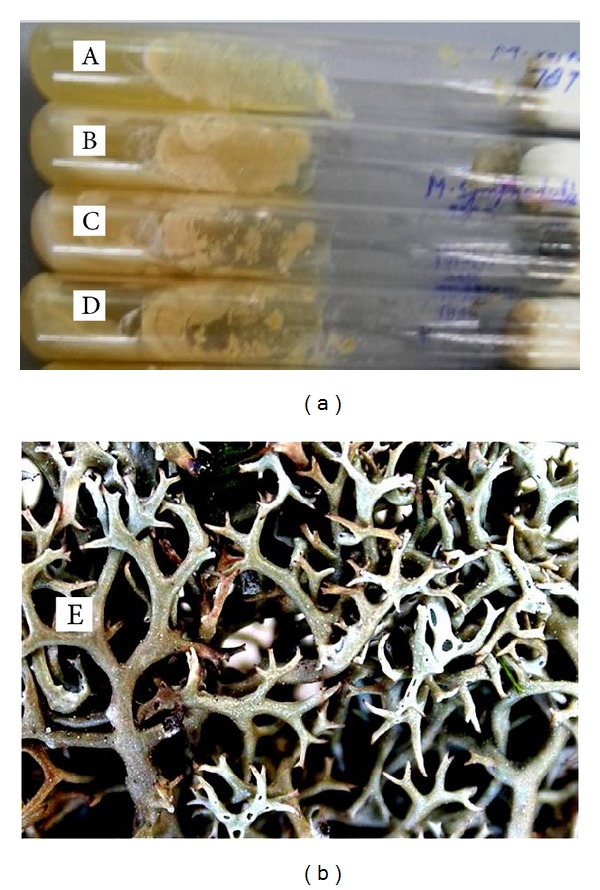
Cultures of *Malassezia* spp.: (A) *Malassezia furfur*, (B) *M. sympodialis,* (C) *M. globosa,* (D) *M. restricta,* and (E) lichen *Cladia aggregata* collected from Sikkim.

10 cultures of unicellular yeast like fungus *Malassezia* spp., namely, *M. furfur, M. globosa, M. restricta, M. sympodialis, M. obtusa, M. sloffiae, M. dermatis, M. yamatoensis, M. nana, and M. japonica,* were obtained from Centraal bureau voor Schimmelcultures (CBS) Fungal Biodiversity Centre, Institute of the Royal Netherlands Academy of Arts and Sciences (KNAW), The Netherlands. *M. furfur* 1878, *M. restricta* 7877, *M. globosa* 7966 and *M. sympodialis* 7222 (Figure 1  (A), (B), (C), and (D)) were selected for this study due to their strong prevalence in causing PV and dandruff in the defined climatic conditions. These cultures were maintained in solid media BPL5 M (patent application number DEL/546/2012) supplemented with powdered milk [18, 19].

### 2.2. Collection of Lichen Material and Preparation of Ethanolic (50% v/v) Extract

The lichen *Cladia aggregata* (Swartz) Nyl. was collected from Sikkim (Figure 1  (E)) and the adjoining areas [20]. The lichen was identified according to the key provided in the Macrolichens of India by Awasthi [21] and further verified by Dr. G. P. Sinha, Scientist, Botanical Survey of India, Central Zone, Allahabad, India. The voucher specimen of air dried lichen material was submitted to the Duthie Herbarium of Department of Botany, University of Allahabad. The air-dried lichen material was washed thoroughly with tap water and then continuous flow of distilled water. After pat drying the sample, 5 grams of lichen sample was weighed and crushed in pestle mortar. It was subjected for cold extraction in 50 mL of ethanolic (50% v/v) solution followed by incubation at 37°C for 24 hours. Subsequently, the extract solution was filtered by Whatman No. 1 filter paper, and filtrate was evaporated in rotary evaporator apparatus at 45–60°C to obtain crude extract. The extract was dried completely and weighed for obtaining percentage yield (0.756 gram, approx. 15%).

### 2.3. Antifungal Susceptibility Testing

The susceptibility of the *Malassezia* spp. was assayed against lichen crude extract using the broth microdilution method recommended by the Clinical and Laboratory Standards Institute (CLSI) [22]. Freshly prepared broth medium BPL5O supplemented with cottonseed oil was used for the assay [18, 19]. Stock solution (50 mg/mL) of extract was prepared in DMSO. In brief, the initial fungal inocula suspension, prepared as per 0.5 McFarland standard (corresponding to a CFU of 1.5 × 10^7^ cell/mL), was inoculated in two-fold serially diluted candidate extract to be tested. Fluconazole, as a synthetic standard, was also subjected to the antifungal assay. The MICs and IC_50_ were obtained by measuring absorbance using spectrophotometer (SpectraMax Plus^384^, Molecular Devices Corporation, USA) at 530 nm, after an incubation of 48 hrs at 35 ± 2°C.

### 2.4. Phylogenetic Treatment of *Malassezia* spp. Studied for Antifungal Assay

Chitin synthase gene (*chs* and/or *chs*-2) responsible for synthesis of chitin (building block of fungal cell wall) was selected for phylogenetic study. This was done because phenolic acids cause initial disruption of cell wall to further act at molecular level. Gene sequences procured from GenBank NCBI database were blasted in the *blastx* programme of NCBI, and amino acid sequences were obtained for the strains (CBS 1878, CBS 7966, CBS 7222, and CBS 7877) used for study [23, 24]. The alignment of the gene sequence (Figure 4) was done by ClustalW analysis, and further phylogeny was constructed (Figure 4) in form of N-J bootstrapped phylogenetic tree [25–27] by MEGA4 software version 4.0 [28]. Some homologous sequences obtained in *blastx *run were also selected randomly for further phylogenetic studies in relation to antifungal susceptibility of *Malassezia* spp. against the extract of *Cladia aggregata* lichen. The phylogenetic tree (Figure 5) was constructed for the *chs* gene along with the translated protein alignment (Figure 6) of the strains studied.

## 3. Results and Discussion

The ethanolic extract of lichen *C. aggregata* (Figure 1  (E)) exhibited an IC_50_ (mg/mL) of 2.51, 0.31, and 0.04 and MIC (mg/mL) of 2.72, 0.63, and 1.28 against *M. furfur, M. globosa, *and* M. sympodialis, *respectively, while no activity was recorded against *M. restricta* (Table 1).

**Figure 2:**
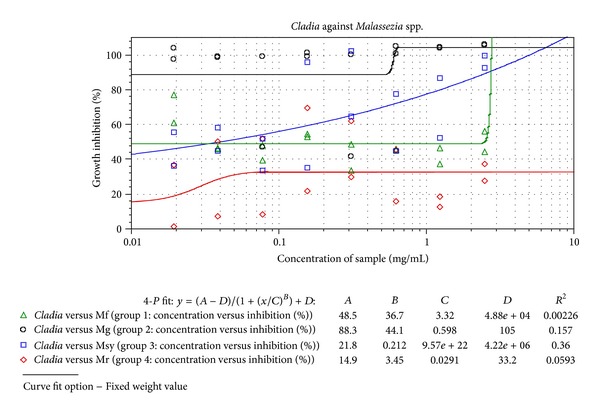
% inhibition curve of *Cladia aggregata* against *Malassezia* spp. (generated by SoftMax Pro using model SpectraMax Plus^384^).

**Figure 3:**
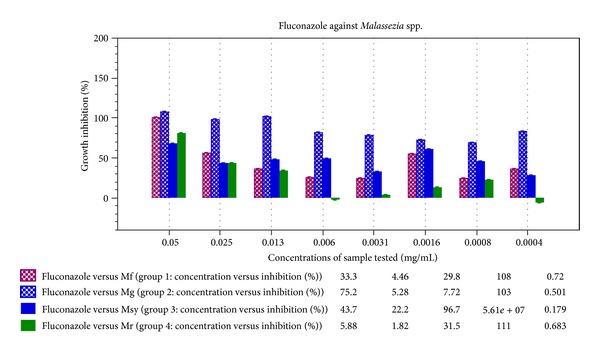
% inhibition curve of fluconazole against *Malassezia* spp. (generated by SoftMax Pro using model SpectraMax Plus^384^).

Fluconazole used as the standard in our study has an IC_50_ (mg/mL) of 0.021, 0.0004, 0.047, and 0.026 and MIC (mg/mL) of 0.034, 0.006, 0.051, and 0.051 against *M. furfur, M. globosa, M. sympodialis* and* M. restricta, *respectively (Table 1). The standard error plot of mean standard deviation (±SD) has been given in the graph calculated by the SoftMax Pro ELISA reader software (Figures 2 and 3).

**Figure 4:**
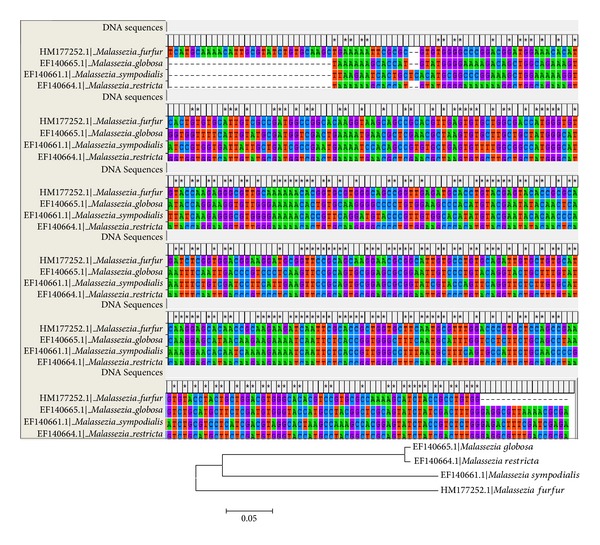
Alignment of the chitin synthase (*chs* gene) of *Malassezia furfur, M. globosa, M. sympodialis* and *M. restricta* by bootstrapped ClustalW program in MEGA 4 (version 4.0) and its phylogenetic tree constructed using the sequences of (*chs* gene) by bootstrapped N-J plot used for the antifungal susceptibility test (sequences of the strains were obtained from NCBI database).

With the point of view of reducing the cost of experimentation for analyzing the variability in MICs against the lichen, *Malassezia *spp. were exposed to phylogenetic analysis by ClustalW analysis and bootstrapping NJ plotting by MEGA 4 (version 4.0). The gene alignment and protein sequences of the *chs* gene obtained from NCBI blast have shown homology in the sequences (Figure 4) and greater confidence level in 1000 bootstrapped N-J plot. The phylogenetic plot also reflected strong susceptibility of *M. globosa* and *M. sympodialis* to *Cladia* extract. It may be considered that more complex species, that is, *M. globosa* and *M. sympodialis, *have more susceptibility to herbal extracts, which was evident from the MICs obtained, that is, 0.63 mg/mL against* M. globosa* and 1.28 mg/mL against *M. sympodialis,* respectively. On the other hand, inhibition of growth of *M. furfur*, which is more primitive, was obtained at 2.72 mg/mL, indicating some resistivity to the herbal extracts. It is noteworthy that *M. globosa* and *M. sympodialis* are frequently isolated pathogenic species from human scalp [29, 30].

**Figure 5:**
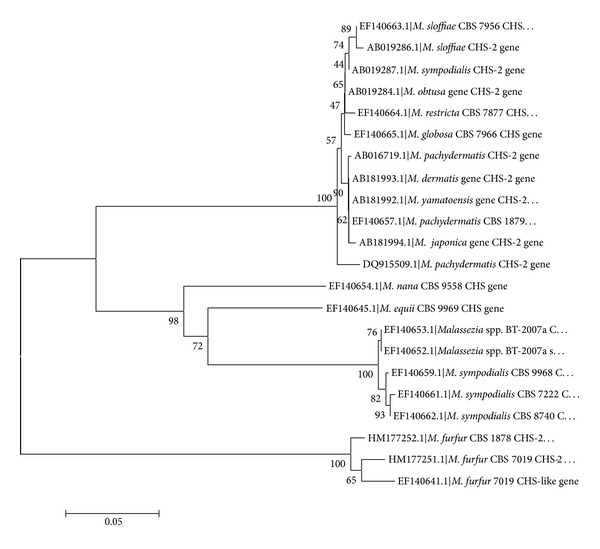
Molecular phylogenetic tree constructed using the sequences of *chs* (chitin synthase) gene of available strains of the genus *Malassezia* with CBS strains used for study (CBS 1878, CBS 7966, CBS 7222 and CBS 7877). The number of branch points represents the percentage of 1000 bootstrapped datasheets showing specific internal branches (sequences of the strains were obtained from NCBI database-accession number given) (constructed by MEGA 4 version 4.0).

**Figure 6:**
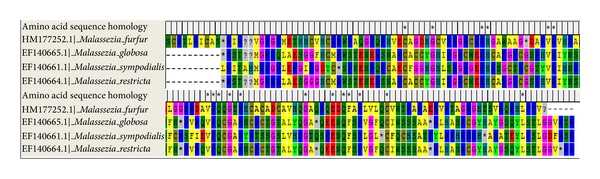
Alignment of the amino acid sequences of *Malassezia furfur* (CBS 1878)*, M. globosa* (CBS 7966)*, M. sympodialis *(CBS 7222) and* M. restricta *(CBS 7877) (constructed by MEGA 4 version 4.0).

This might be due to the homology in the chitin synthase enzyme translated by *chs* gene (Figure 6). The wall structure of the fungi can be considered as one factor. The more primitive *M. furfur* has a stouter wall, which restricts the action of antifungal agent, whereas *M. globosa *and* M. sympodialis* have shown more susceptibility to the agent. Moreover, on the basis of molecular phylogeny of various available strains of *Malassezia *along with CBS standard strains used for our study (Figure 5), the effectiveness of the extract was in strict accordance to the closely related *Malassezia* spp.; it can be conceived that the *Cladia* extract will also be effective against other anthropophilic and zoophilic spp., namely, *M. pachydermatis, M. japonica, M. yamatoensis *and* M. equii. *The *C. aggregata*, along with *Usnea baileyi *and* Everniastrum nepalense*, has been found active against multidrug resistant *Staphylococcus aureus *[31, 32]. Established results on the antifungal activity of *Everniastrum cirrhatum* with minimum fungicidal concentration (MFC) of as low as 60 *μ*L/mL against human pathogenic fungi (dermatophytes), namely, *Epidermophyton floccosum, Microsporum gypseum, M. canis, M. audounii, Trichophyton rubrum, T. mentagrophytes, T. violaceum, *and* T. tonsurans,* have also been reported in the past [33]. *Heterodermia leucomelos *was also found effective against human as well as plant pathogenic fungi [34]. Some macrolichens extracts, namely, *Parmelia tinctorum, Ramalina *sp., *Teloschistes flavicans, *and *Usnea undulata*, were tested and found effective against some pathogenic fungi [35]. Broad spectrum antifungal properties at 80 *μ*L/mL were evident in the aqueous extract of *Parmelia cirrhatum* against some human and plant pathogens [36]. The phenolic compounds and their derivatives in lichen have been proved to be detrimental for pathogenic microbial fauna. These substances generally acidify the microbial cell wall and consequently, cause cytoplasm membrane rupture, inactivate or immobilize the enzymes, and interfere with physiological functions such as electrons transport and oxidative phosphorylation [37–39]. A number of higher plants have been reported effective against dandruff causing *Malassezia *[40], but none have comparable potentiality with lichens against *Malassezia*. To the best of our knowledge, the activity of lichen *C. aggregata* against *Malassezia furfur, M. globosa *and* M. sympodialis* is reported for the first time and will have potential for the development of cosmaceuticals.

## 4. Conclusion

The present finding creates an interest in the exploration of lichens for novel antimicrobials. The nontoxic nature of herbal medicines complements conventional treatment and excels over the synthetic drugs such as fluconazole, which are effective but come with considerable side effects and have high disease reoccurrence rate. Moreover, the bioprospection should not be limited to mere exploration of the novel antimicrobials but should lead to development of the formulation after successful multicentral topical testing, pharmacological, and toxicological investigations. To the best of our knowledge, this is the first report for the anti-*Malassezia* property of lichen *Cladia aggregata* (Swartz) Nyl. against the three most prevalent PV and dandruff causing mycoflora, namely, *M. globosa, M. furfur* and* M. sympodialis. *The prediction of the susceptibility of the pathogenic fungus towards active compounds based on their phylogenetic position is a novel approach. Thus, the present findings strongly support the potentiality of the lichen *C. aggregata* as a useful herbal cosmaceutical after successful topical testing, which is in progress.

## Figures and Tables

**Table 1:** Antifungal activity of 50% ethanolic extract of *Cladia aggregata* (Swartz) Nyl. compared with synthetic fluconazole against the *Malassezia* spp.

Selected pathogens	Antifungal activity			
	Ethanolic extract *Cladia aggregata* (Swartz) Nyl.		Fluconazole	
	IC_50 _(mg/mL)	MIC (mg/mL)	IC_50 _(mg/mL)	MIC (mg/mL)
*M. furfur *	2.51	2.72	0.021	0.034
*M. globosa *	0.31	0.63	0.0004	0.006
*M. sympodialis *	0.04	1.28	0.047	0.051
*M. restricta *	No activity	No activity	0.026	0.051
